# An automatic method for the determination of bromide in water

**DOI:** 10.1155/S1463924680000417

**Published:** 1980

**Authors:** R. E. D. Moxon, E. J. Dixon

**Affiliations:** 1 Food and Nutrition Division Laboratory of the Government Chemist Cornwall House Stamford Street London SE1 9NQ UK; 2 Department of Health and Social Security 14 Russell Square London WC13 5EP UK


### An automatic method for the determination of bromide in water

**R.E.D. Moxon**
*Food and Nutrition Division, Laboratory of the Government Chemist, Cornwall House, Stamford Street, London SE1 9NQ.*
**E.J. Dixon**
*Department of Health and Social Security, 14 Russell Square, London WC13 5EP.*

### Introduction

A project to develop and test methods for the determination of bromide species in fresh and potable waters, initiated and funded by the Department of the Environment, was undertaken at the Laboratory of the Government Chemist.

A literature search revealed three methods that had a sensitivity which could meet the requirements of the Department of the Environment. The method described by Fishman and Skougstad [1] is based on the catalytic effect of the bromide ion on the oxidation of iodine to iodate by permanganate . The excess iodine is extracted with carbon tetrachloride and measured spectrophotometrically. This method claims a detection limit of 1$\mu$g per litre. Tamarchenko [2] described a method based on the oxidation of bromide to bromate which is reduced by excess bromide to bromine in acid solution. The bromine decolourises methyl orange which is measured spectrophotometrically. This method claims a detection limit of 20 $\mu$g of bromide per litre. Archimbaud and Bertrand [3] utilised a Technicon AutoAnalyzer system based on the synthesis of tetrabromosulphonphthalein purple at pH 4.6 from phenosulphonphthalein and bromide previously oxidised to bromine by chloramine T. This method claims a detection limit of 20 $\mu$g of bromide per litre.

Of the three methods, the one developed by Fishman and Skougstad was selected for further investigation as it was the most sensitive and was not subject to interferences from many other ions at concentrations found in natural waters.

### Experimental

Although the method gave a satisfactory calibration curve for bromide standard solutions of 20 to 100 $\mu$g per litre it was found to be critically affected by a) the time of oxidation; b) the temperature of the oxidation; and c) the expertise involved in adding reagents and shaking separators at accurately timed intervals. In order to control and standardise these conditions more closely, to enable greater productivity to be achieved and to protect the operator from frequent contact with carbon tetrachloride it was decided to automate the method using a Technicon Mark I AutoAnalyzer system. The initial procedure using standard Technicon components reproduced the chemistry of the manual method almost exactly but revealed several problems which are described below.

### Problems encountered during automation of the manual method

1. Iodine was deposited on the transmission tubing and on the pump tube used for the potassium iodide/sulphuric acid reagent. This was overcome by introducing the potassium iodide and sulphuric acid separately and allowing them to mix in a glass coil within the automated system.

2. Deposits of manganese dioxide, which altered the sensitivity of the method, started to form in the extractor coil and separator trap after a couple of hours continuous use. This was overcome by pumping a solution of 5% sodium oxalate through the system for two minutes every two hours to reduce the manganese dioxide and clean the system. A working temperature of 0°C also helped to minimise manganese dioxide formation as well as slowing the time of oxidation of iodine to iodate to several minutes, thus reducing the relative error involved in measuring the exact time of the reaction.

3. Two different extractor coils were tried and found to be unsuitable as the organic phase did not pass through regularly and this led to broad irregularly shaped peaks on the chart recorder. A Technicon 7 turn mixing coil of 2.4 mm internal diameter was packed with glass beads of 1 mm diameter and found to give a faster throughput and more regular peaks.

4. Technicon separator traps type BO and B4 were both found to be unsuitable. Their large volume resulted in considerable peak broadening and necessitated a long wash interval between samples. A Technicon C8 debubbler, as shown in Figure 1, was therefore used to separate the phases. The strip of phase-separating paper was found to be essential for preventing droplets of the aqueous phase from entering the flowcell, and to aid the coalescence of droplets of carbon tetrachloride. A teflon insert (Technicon part no 021 0002 02) inserted into a C8 debubbler leading from the arm carrying the sample stream into the lower leg carrying the solvent phase was found to be a suitable alternative.

5. Solvaflex pump tubes were found to be unsuitable for pumping carbon tetrachloride for long periods of time and therefore the water displacement method shown in Figure 2 was used. Introduction of carbon tetrachloride into the system in a regular stream of droplets was found to be essential for obtaining a regular baseline and was best achieved by using a Technicon A6 fitting with platinum insert and either polythene or Acidflex tubing (internal diameter 0.030 inches) to connect the displacement flask to the manifold.

6. A regular interference associated with the sampler was removed by eliminating the bubble introduced during the sampling action.

7. Small air bubbles appeared intermittently in the flowcell, disturbing the baseline. These were removed by introducing a 10 turn mixing coil between the extractor coil and the separator. This gave any air which had dissolved in the carbon tetrachloride in the extractor coil time to come out of solution before entering the flowcell.

The final manifold which is shown in Figure 2 is suitable for the analysis of bromide in water over a range of 5 to 100 $\mu$g/l.

### Reagents

All chemicals used are of analytical reagent grade quality.

**Potassium bromide solution (100 mg $l^{-1}$ Br)**
Dissolve 0.149 g of potassium bromide in distilled water and dilute to 1 litre in a volumetric flask. (Stable for one month).

**Potassium bromide solution (5 mg $l^{-1}$ Br)**
Dilute 50ml of the potassium bromide solution (100mg $l^{-1}$ Br) with distilled water to one litre in a volumetric flask. (Stable for one month).

**Potassium bromide solution (100 $\mu$g $l^{-1}$ Br)**
Dilute 20 ml of potassium bromide solution (5 mg $l^{-1}$ Br) with distilled water to one litre in a volumetric flask. (Stable for one month).

Dilute 80, 60, 40, 20, 10, 5, 0 ml of potassium bromide solution (100 $\mu$g $l^{-1}$ Br) with distilled water to 100 ml in volumetric flasks. These are the working standards. Store in glass bottles away from light and prepare freshly at two week intervals.

**Sodium chloride solution (500 mg $l^{-1}$ Cl)**
Dissolve 0.824 g of sodium chloride in distilled water and dilute to 1 litre in a volumetric flask.

Dilute 10, 8, 6, 4, 2 ml of sodium chloride solution (500mg $l^{-1}$ Cl) with distilled water to 100 ml in volumetric flasks. These are the chloride working standards. Store in polythene bottles and prepare freshly at two week intervals.

**Potassium permanganate solution (0.632 percent m/V)**
Dissolve 6.32g of potassium permanganate in distilled water and dilute to one litre. Store in an amber glass bottle in a refrigerator, and filter through a Whatman 541 filter paper before use.

**Potassium iodide solution (0.131 percent m/V)**
Dissolve 1.31 g of potassium iodide, previously dried in a desiccator, in distilled water and dilute to one litre.

**Sulphuric acid solution (17.5 percent V/V)**
Add 175 ml of concentrated sulphuric acid (Sp Gr 1.84) slowly, and with stirring, to 600 ml of distilled water. Allow to cool and dilute to one litre.

### Apparatus for bromide determination

The Technicon AutoAnalyser system used consisted of a powerpack, proportioning pump, recorder, colorimeter and range expander of the Mark I type, and a Sampler II. The colorimeter was used with a 15 mm flowcell and 520 nm filters. The sampler was used with a 20 samples per hour (1/2) cam. A Technicon 7 turn mixing coil of 2.4 mm internal diameter packed with 1 mm diameter glass beads and restricted at both ends to prevent the beads from falling out was used as an extractor coil. To separate the organic from the aqueous phase a Technicon CB debubbler fitted with a strip of Whatman No 1 phase-separating paper, as shown in Figure 2, was used. The system was kept at 0° C ± 0.3 using an ice bath insulated externally with polystyrene and two 500 ml flasks were used for the water displacement of carbon tetrachloride. All connections were made with glass tubing unless otherwise indicated on the flow diagram (Figure 1) and Solvaflex sleeving was used to butt glass tubing together. The Technicon equipment was operated and maintained in accordance with the procedure described in the Technicon Mark I Assembly and Operating Instruction Manual.

### Apparatus for chloride determination

The method used for chloride determination in this paper was the Technicon AutoAnalyser II Industrial Method 73–71E; 'chloride in water' (published by Technicon Industrial Systems, Tarrytown NY 10591). The results were obtained using a Technicon AutoAnalyser II system, and compared favourably with those obtained using a Corning EEL model 921 chloride electrode (Evans Electroselenium, Halstead, Essex, UK) operated according to the makers instructions.

### Procedure

Set up the manifold system as shown in the flow diagram (Figure 1). Fill the ice bath with ice pieces and then fill

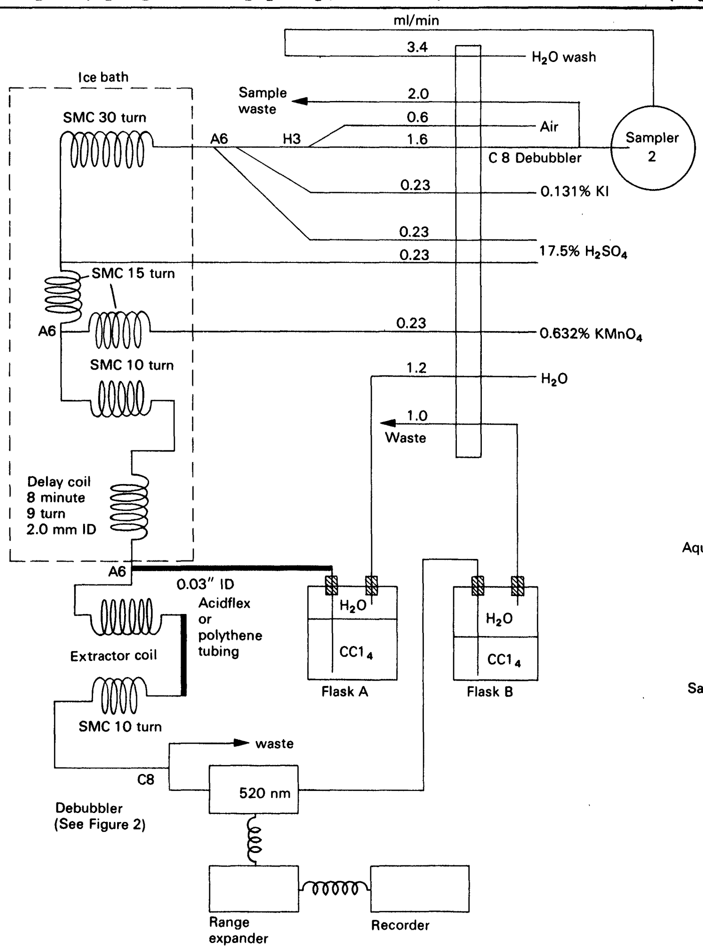

*Figure 1. (left) Flow diagram for AutoAnalyser I manifold developed for the determination of bromide in water.*

*Figure 2. (below) Arrangement of Technicon C8 debubbler for use as a phase separator.*

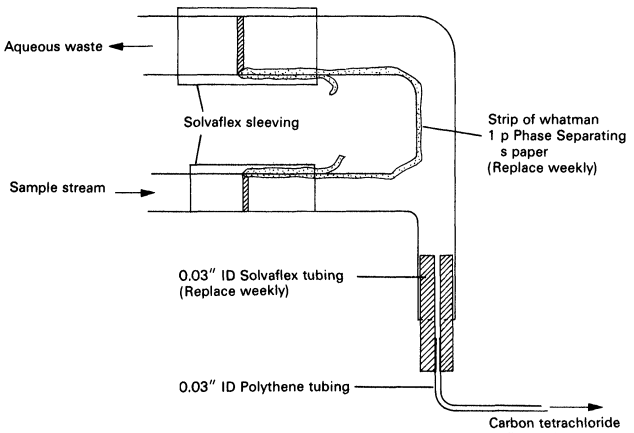

with water. Check the temperature once per hour and maintain at 0°C ± 0.3. Fill displacement flask A almost to the top with carbon tetrachloride and flask B with water. Use distilled water to completely fill both flasks and connecting tubes to make sure that all air is removed and to prevent surging. Place the sulphuric acid, potassium iodide and potassium permanganate reagents in the ice bath. Pump distilled water through all reagent and sample lines until a regular bubble pattern is maintained and carbon tetrachloride is passing through the flowcell free from any air bubbles or droplets of aqueous phase. Pump the reagents through the system using water in the sample line and the reaction will occur with a maximum of iodine being extracted into carbon tetrachloride, giving it a purple colour. Allow twenty minutes for the extraction and separation to stabilise and then use the Operator's Instruction Manual to adjust the colorimeter and range expander to give a full scale deflection of 80 percent for the top working standard. Load the sample tray with a set of bromide working standards followed by 20 samples interspersed with a working standard every fifth sample. Complete the series with another set of bromide working standards and then pump sodium oxalate solution through the sample line to remove any deposits of manganese dioxide. Load the sample tray with a set of bromide working standards followed by a set of chloride working standards in triplicate and complete the series with a set of bromide working standards. The chloride standards should be run each time a new set of bromide working standards are prepared. Determine the chloride concentration of the samples using a suitable method having an accuracy and precision and detection limit of 1 mg $l^{-1}$.

### Calculation a) Plot a calibration curve of the mean bromide standard peak heights against their respective bromide concentrations. The apparent bromide concentration of the sample (A $\mu g$ $l^{-1}$) which represents the true bromide concentration plus any additional response due to chloride interference is obtained by comparing its peak height with the calibration curve.

b) Obtain the apparent bromide concentration of the working chloride solutions by comparing their peak heights with the bromide calibration curve.

c) Plot a calibration graph of the chloride concentrations against their corresponding bromide concentrations. This should be a straight line with a gradient G ($\mu g$ $l^{-1}$ Br per mg $l^{-1}$ Cl).

d) Determine the 'true' bromide value of the sample (X $\mu g$ $l^{-1}$ Br) by subtracting the bromide concentration caused by chloride interference from the apparent bromide concentration of the sample (A) according to the following formula.

$$X\ (\mu g\ l^{-1}\ Br) = A - (D \times G)$$

where D is the chloride concentration of the sample solution.

### Results and discussion

### Effect of interferences

Fishman and Skougstad [1] investigated the effects of temperature, concentration of reactants and interferences on the method. The effects of some interfering ions were determined by the present authors and the results are shown in Table 1. The mean and maximum values for the concentration of elements in drinking water are those reported by Zoetman and Brinkmann [4].

The effects of interferences generally agreed with those reported by Fishman and Skougstad [1], and any minor differences were probably due to the salts used to add the interfering elements. For most drinking waters the effects of interferences except for that of chloride will be negligible. Some mineral waters and contaminated drinking waters can contain elements such as iron and manganese in amounts which could give substantial interference and these could be diluted to a level where the interference was removed.

### The effect of chloride

Chloride was found to interfere positively in the proposed automated system at a concentration well below that found in most waters. Fishman and Skougstad did not include chloride in the list of possible interfering ions for the manual method. At first, bromide contamination of the sodium chloride used to test chloride interference was suspected, but 50 mg $l^{-1}$ chloride solutions made from three AR chloride salts and Analar HCL all gave the same chart response. Several approaches were tried to obviate the effects of chloride by chemical means, such as varying the pH of the reaction mixture, using weaker oxidising reagents and introducing chloride into the manifold at a concentration that would make that present in waters insignificant. All these proved unsuccessful. It was noted that the apparent bromide concentrations of chloride standard solutions obtained by comparing the chloride solution peak heights against the bromide calibration curve increased linearly up to 50mg $l^{-1}$ Cl, and that the results were precise and reproducible (Table 2).

The gradient of the straight line obtained by plotting chloride concentration against the corresponding bromide concentration was 1.31 $\mu g$ $l^{-1}$ of bromide per mg $l^{-1}$ of chloride.

A series of bromide solutions spiked with known amounts of chloride were run by the proposed method. The bromide concentration corresponding to the chloride 'interference' was calculated using the gradient of the straight line described in Table 2 and this was subtracted from the apparent bromide concentration of the solution to give a calculated value for the true bromide concentration. The results are shown in

**Table 1. The effect of added ions on the determination of bromide in a 50 $\mu g$ per litre standard solution**

| Ions added | Added as | Concentration of ion $\mu g$ $l^{-1}$ | Concentration in drinking waters $\mu g$ $l^{-1}$ | | *Bromide found $\mu g$ $l^{-1}$ |
|---|---|---|---|---|---|
| | | | mean | maximum | |
| $Fe^{3+}$ | $Fe(NO_3)_3 9H_2O$ | 2000 | 130 | 1500 | 50 |
| " | " | 3000 | " | " | 58 |
| $I^-$ | KI | 10000 | – | – | 50 |
| " | " | 20000 | – | – | 47 |
| $Mn^{2+}$ | $MnSO_4.4H_2O$ | 1000 | 24 | 60 | 53 |
| " | " | 3000 | " | " | 60 |
| $S_2O_3^{2-}$ | $Na_2S_2O_3.5H_2O$ | 1000 | – | – | 54 |
| $SO_4^{2-}$ | $Na_2SO_4$ | 1000 | – | – | 51 |
| Cl gas | | | – | – | 0 |
| $Zn^{2+}$ | $Zn(NO_3)_2.6H_2O$ | 2000 | 110 | 690 | 50 |
| $Ag^+$ | $AgNO_3$ | 1000 | – | – | 50 |
| $Sr^{2+}$ | $Sr(NO_3)_2$ | 1000 | 100 | – | 50 |
| $Li^{2+}$ | $Li(NO_3)_2$ | 1000 | 8 | 10 | 50 |
| $Cu^{2+}$ | $Cu(NO_3)_2.3H_2O$ | 1000 | 50 | 360 | 50 |
| $F^-$ | NaF | 1000 | – | – | 50 |
| $Pb^{2+}$ | $Pb(NO_3)_2$ | 1000 | 15 | 46 | 50 |
| $Cl^-$ | NaCl | 10000 | $52 \times 10^3$ | $245 \times 10^3$ | 63 |
| Br | $cHBr_3$ | 5000 | – | – | 50 |

**The bromide values shown are a mean of three determinations.*

**Table 2. Apparent bromide concentration of chloride solutions**

| Chloride concentration (mg $l^{-1}$) | Apparent bromide concentration ($\mu g$ $l^{-1}$) (mean of 6 determinations) | Coefficient of variation |
|---|---|---|
| 50 | 65.5 | 3.2 |
| 40 | 52.2 | 3.6 |
| 30 | 39.6 | 5.0 |
| 20 | 25.6 | 3.9 |
| 10 | 12.6 | 5.6 |

**Table 3. Calculated concentrations of bromide in solutions spiked with known amounts of chloride**

| Bromide concentration $\mu g\ l^{-1}$ | Chloride concentration D mg $l^{-1}$ | Apparent bromide concentration A $\mu g\ l^{-1}$* | Bromide equivalent of chloride spike D x G $\mu g\ l^{-1}$ | Calculated bromide concentration A-(D x G) $\mu g\ l^{-1}$ | % bromide found |
|---|---|---|---|---|---|
| 50 | 10 | 62.7 | 13.1 | 49.6 | 99 |
| 50 | 20 | 74.5 | 26.2 | 48.3 | 97 |
| 50 | 30 | 91.0 | 39.3 | 51.7 | 103 |
| 20 | 20 | 46.8 | 26.2 | 20.6 | 103 |
| 20 | 30 | 61.0 | 39.3 | 21.7 | 108 |
| 20 | 40 | 72.7 | 52.4 | 20.3 | 101 |

**mean of 4 determinations (the letters are those used in the section 'calculation'.)*

Table 3. As the bromide concentration corrected for the effect of chloride interference was so near to the actual value, it was felt justifiable to apply this approach to water samples, as most laboratories engaged in water analysis determine chloride concentration as a matter of routine. The extent to which chloride interferes in the determination of bromide by the proposed method is shown in a range of UK drinking waters. The results are summarised in Table 4.

### The effect of bromate

Solutions of potassium bromide (A) and potassium bromate (B) each containing 100 $\mu$g of bromine per litre both gave the same response on the chart recorder. A series of solutions were prepared by mixing A and B in the following proportions:– 1:3, 1:2, 1:1, 2:1, 3:1. These all gave the same response on the chart recorder indicating that bromide and bromate have similar catalytic activity.

### Precision and accuracy of the method

To obtain a measure of the precision of the method, drinking waters from different areas in the United Kingdom were analysed on each of four consecutive days. The results are summarised in Table 5a and b where variations in the peak heights of working standard solutions and individual bromide results are expressed by the standard deviation from the mean. This shows that the general precision of the method is of the order of six percent.

As no certificated water sample against which to test the accuracy of the method could be found, and as no suitable reference method was available, an indication of the accuracy of the method was obtained by adding known amounts of bromide to diluted water samples which had been previously analysed for bromide. The final bromide concentrations shown are a mean of 4 separate determinations. The amount of bromide found ranged from 98 to 103 percent of the amount calculated to be present in the solution. The results are summarised in Table 6.

### The limit of detection

The limit of detection was taken to be where the signal level was three times greater than the noise level of the baseline. This gave a limit of detection of 4 $\mu$g of bromide per litre. Should greater sensitivity be required, water samples could be reduced in volume by evaporation, or alternatively the temperature at which the reaction takes place could be increased, thereby increasing the sensitivity.

### Conclusion

The automated method has an accuracy, and precision comparable with the manual method. Once the automated equipment has been assembled and tested, an assistant with little training can analyse a large number of samples very quickly. Results to a similar standard can only be obtained manually at a much slower rate by employing a skilled operator.

*(Crown copyright)*

ACKNOWLEDGEMENTS

The authors thank the Government Chemist and the Department of the Environment for permission to publish this paper.

**Table 4. The extent of chloride interference expressed as a percentage of the apparent bromide concentration in a range of UK waters**

| Town | Apparent bromide concentration $\mu g\ l^{-1}$ | Chloride concentration (by electrode) mg $l^{-1}$ | Bromide contribution from chloride $\mu g\ l^{-1}$ | True bromide concentration $\mu g\ l^{-1}$ | % Chloride interference |
|---|---|---|---|---|---|
| Newcastle | 29 | 9 | 12 | 17 | 41 |
| Birmingham | 15 | 5 | 7 | 8 | 47 |
| Oakhampton | 35 | 10 | 13 | 22 | 37 |
| Evensford | 145 | 25 | 33 | 112 | 23 |
| Beeston | 690 | 100 | 131 | 560 | 19 |
| Pewsey | 50 | 12 | 16 | 34 | 32 |
| Bicester | 115 | 10 | 13 | 102 | 11 |
| Dover | 122 | 25 | 33 | 89 | 27 |
| Hereford | 40 | 15 | 20 | 20 | 50 |
| Oban | 23 | 15 | 20 | 3 | 87 |
| Milton Keynes | 210 | 62 | 81 | 129 | 39 |
| Invarary | 60 | 11 | 14 | 46 | 23 |

**Table 5. Variations in peak heights of standard solutions and bromide concentrations of samples run on each of 4 consecutive days.**

Standard solution peak heights (percent full scale deflection)

| Concentration $\mu g\ l^{-1}$ | Mean peak height | Standard deviation | Coefficient of variation |
|---|---|---|---|
| 100 | 67.0 | 1.5 | 2.2 |
| 80 | 58.8 | 1.9 | 3.2 |
| 60 | 48.6 | 1.2 | 2.4 |
| 40 | 35.7 | 2.0 | 5.5 |
| 20 | 20.8 | 1.1 | 5.3 |
| 10 | 11.7 | 0.9 | 7.3 |
| 5 | 6.5 | 0.5 | 7.0 |
| **Sample** | **Mean bromide concentration $\mu g\ l^{-1}$** | **Standard deviation** | **Coefficient of variation** |
| Reading | 93 | 8.5 | 9.1 |
| Norwich | 97 | 5.3 | 5.5 |
| London | 397 | 3.6 | 9.2 |
| Lincoln | 205 | 9.2 | 4.5 |
| Catterick | 36 | 1.8 | 5.1 |
| Chester | 57 | 3.1 | 5.4 |
| Lulworth | 94 | 3.6 | 3.8 |

**Table 6. Recovery of added bromide from water samples**

| Sample | Bromide present in diluted water $\mu g\ l^{-1}$ | Bromide added $\mu g\ l^{-1}$ | Calculated total $\mu g\ l^{-1}$ | Bromide found $\mu g\ l^{-1}$ | % recovery |
|---|---|---|---|---|---|
| Reading | 19 | 40 | 59 | 60 | 102 |
| Norwich | 20 | 40 | 60 | 61 | 102 |
| London | 16 | 40 | 56 | 56 | 100 |
| Lincoln | 21 | 40 | 61 | 60 | 98 |
| Catterick | 14 | 40 | 54 | 54 | 100 |
| Chester | 11 | 40 | 51 | 52 | 102 |
| Lulworth | 19 | 20 | 39 | 40 | 103 |

REFERENCES

[1] Fishman, M.J. and Skougstad, M.W., *Analytical Chemistry*, 1963, **35**, 146.

[2] Tamarchenko, L.M., *Gigiyene*, 1975, 1, 80.

[3] Archimbaud, M. and Bertrand, M.R., *Chimie Analytique*, 1970, **52**, (5), 531.

[4] Zoetman, B.C.J. and Brinkmann, F.J.J., "Hardness of Drinking Water and Public Health", Proceedings of the European Scientific Colloquum, Luxembourg, May 1975, Pergamon Press Ltd., 1976, 173–211, Oxford, UK.

